# Improving Patient Experience and Flow in the Referral Pathway with Multi-Disciplinary Team Interventions (MDT) in a Community Mental Health Team in Newham: A Quality Improvement Project

**DOI:** 10.1192/bjo.2025.10438

**Published:** 2025-06-20

**Authors:** Priyanka Tharian, Gokce Saridogan, Michelle Heffernan, Ertugrul Saral, Hanan Saleh

**Affiliations:** East London Foundation Trust, London, United Kingdom

## Abstract

**Aims:** The goal of this quality improvement (QI) project is to enhance the patient journey and flow through the referral pathway into our sub-team in Newham’s community integrated mental health service (CIMHS). The key objectives are to reduce waiting times for appointments, and reduce the high non-attendance rates by improving multi-disciplinary team (MDT) interventions from the point of referral through to review by a psychiatrist. We also aim to streamline the triage system to ensure clearer criteria for medical reviews.

**Methods:** Our methods include 6 major interventions:

Data will be collected from the admin team to understand “did not attend” (DNA) trends and address underlying factors.

An occupational therapy group programme will be used to support patients waiting for medical appointments.

Collaborating with the psychology department and addressing the lack of team psychologists will be explored for psychological support.

MDT members will use a standardised quality of life questionnaire, to screen patients’ needs, offering appropriate interventions.

Through MDT meetings, actively managing the caseload and ensuring timely discharge of stable patients or those who no longer require the service.

Enhancing the quality of primary care referrals with clearer guidelines to improve the triage process incorporating a standardised new referral form.

**Results:** Within our caseload of 285 patients, we reviewed current waiting times for appointments in our team from the time of referral. The average waiting time for a medical review was 54 days, and for a non-medical appointment 38 days. These waiting times have risen in the last few months and we will analyse the factors behind this as part of the project. Over the last three months, there were two comments on patient feedback forms reporting that the waiting time for appointments was too long, with similar informal verbal feedback from other patients. We will review the patient reported experience measure forms’ (PREM) feedback, following the implementation of the above MDT interventions after 3 months.

**Conclusion:** Our team needs to reduce the long waiting times for appointments and address high DNA rates to improve the efficiency of the service, while enhancing the patient experience. Currently, many new referrals are directed to medical reviews, but MDT involvement could offer earlier and more holistic interventions, addressing quality of life domains. We can promote discharge to primary care as a positive step towards recovery, with the option to opt back in, if there’s a need in future.

